# Cerebral Autoregulation Evidenced by Synchronized Low Frequency Oscillations in Blood Pressure and Resting-State fMRI

**DOI:** 10.3389/fnins.2019.00433

**Published:** 2019-05-07

**Authors:** Joseph R. Whittaker, Ian D. Driver, Marcello Venzi, Molly G. Bright, Kevin Murphy

**Affiliations:** ^1^Cardiff University Brain Research Imaging Centre (CUBRIC), School of Physics and Astronomy, Cardiff University, Cardiff, United Kingdom; ^2^CUBRIC, School of Psychology, Cardiff University, Cardiff, United Kingdom; ^3^Department of Physical Therapy and Human Movement Sciences, Feinberg School of Medicine, Northwestern University, Chicago, IL, United States

**Keywords:** cerebral autoregulation, resting-state fMRI, blood pressure, cerebral physiology, LFO, BOLD, CBF

## Abstract

Resting-state functional magnetic resonance imaging (rs-fMRI) is a widely used technique for mapping the brain’s functional architecture, so delineating the main sources of variance comprising the signal is crucial. Low frequency oscillations (LFO) that are not of neural origin, but which are driven by mechanisms related to cerebral autoregulation (CA), are present in the blood-oxygenation-level-dependent (BOLD) signal within the rs-fMRI frequency band. In this study we use a MR compatible device (Caretaker, Biopac) to obtain a non-invasive estimate of beat-to-beat mean arterial pressure (MAP) fluctuations concurrently with rs-fMRI at 3T. Healthy adult subjects (*n* = 9; 5 male) completed two 20-min rs-fMRI scans. MAP fluctuations were decomposed into different frequency scales using a discrete wavelet transform, and oscillations at approximately 0.1 Hz show a high degree of spatially structured correlations with matched frequency fMRI fluctuations. On average across subjects, MAP fluctuations at this scale of the wavelet decomposition explain ∼2.2% of matched frequency fMRI signal variance. Additionally, a simultaneous multi-slice multi-echo acquisition was used to collect 10-min rs-fMRI at three echo times at 7T in a separate group of healthy adults (*n* = 5; 5 male). Multiple echo times were used to estimate the R_2_^∗^ decay at every time point, and MAP was shown to strongly correlate with this signal, which suggests a purely BOLD (i.e., blood flow related) origin. This study demonstrates that there is a significant component of the BOLD signal that has a systemic physiological origin, and highlights the fact that not all localized BOLD signal changes necessarily reflect blood flow supporting local neural activity. Instead, these data show that a proportion of BOLD signal fluctuations in rs-fMRI are due to localized control of blood flow that is independent of local neural activity, most likely reflecting more general systemic autoregulatory processes. Thus, fMRI is a promising tool for studying flow changes associated with cerebral autoregulation with high spatial resolution.

## Introduction

Functional connectivity in the brain can be assessed with blood-oxygenation-level-dependent (BOLD) functional magnetic resonance imaging (fMRI). The source of BOLD contrast is the difference in magnetic susceptibility between oxy- and deoxyhemoglobin, which has an effect on apparent transverse relaxation (R_2_^∗^), and thus imparts sensitivity to blood oxygenation in the MR signal (Buxton, 2013). Neurovascular coupling (NVC) allows brain activity to be mapped using BOLD fMRI, because localized increases in cerebral blood flow (CBF), which are proportionally larger than changes in oxygen metabolism, cause increases in local venous blood oxygenation. An implicit assumption that predicates BOLD fMRI as a tool for mapping neural activity in the brain is that NVC related changes in CBF are the predominant source of signal variance. There are, however, other mechanisms besides NVC that regulate CBF, such as arterial blood gas concentration, particularly carbon dioxide (CO_2_), which is a potent vasodilator with a strong effect on CBF (Battisti-Charbonney et al., 2011). Furthermore, systemic control of the brain’s blood supply is governed by numerous homeostatic mechanisms that are broadly defined as cerebral autoregulation (CA) (Willie et al., 2014), the theoretical process that modulates cerebrovascular resistance to ensure CBF is kept at a sufficient level in the face of transient changes in systemic haemodynamics (e.g., blood pressure and cardiac output).

Understanding non-neuronal sources of variance in CBF fluctuations is especially important with regard to resting state fMRI (rs-fMRI) paradigms for two reasons. Firstly, unlike traditional task based paradigms for which the timing and duration of evoked BOLD signal changes is known a priori, the timing of spontaneous neural fluctuations can’t be assumed, meaning non-neuronal effects can’t be mitigated as they are in task based designs by averaging over trials. Secondly, the low frequency range (∼0.01–0.1 Hz) over which functional connectivity is observed overlaps with the spectrum at which other systemic physiological effects occur (Murphy et al., 2013). Spontaneous fluctuations in breathing cause cerebrovascular reactivity (CVR) to CO_2_ to manifest as low frequency (<0.05 Hz) oscillations in the BOLD signal (Wise et al., 2004), and endogenous fluctuations (<0.1 Hz) in vascular tone have been reported in various different vascular beds across multiple species (Nilsson and Aalkjaer, 2003). Recently, low frequency oscillations (LFO) of a systemic origin have been observed by correlating fMRI signals with functional near-infrared spectroscopy (fNIRS) measures of peripheral haemodynamics (Tong and Frederick, 2010; Tong et al., 2012). Intriguingly, these systemic LFOs appear to propagate throughout the brain with spatially structured temporal delays (Erdogan et al., 2016).

Arterial blood pressure (ABP) is dynamic over multiple time scales, including at a beat-to-beat level, and so is likely to contribute significantly to fluctuations in CBF. Transcranial Doppler ultrasound (TCD) studies have consistently demonstrated how ABP fluctuations modulate cerebral blood flow velocity (CBFV) in large intracranial arteries (Aaslid et al., 1989; Zhang et al., 1998), and that they account for a considerable proportion of the total variance, approximately 60% of the total predictive power of CBFV fluctuations in right middle cerebral artery (MCA) (Mitsis et al., 2004). Evidence for how blood pressure dynamics affect fMRI fluctuations is scarce, mostly limited to animal studies on the relationship between transient changes and evoked neural responses (Wang et al., 2006; Qiao et al., 2007; Uchida et al., 2017). Similar to fMRI, ABP time series have a 1/f power spectrum, but also show distinct oscillations (∼0.1 Hz in humans) known as Mayer waves (Mayer, 1876), which are independent of respiration and tightly coupled to efferent sympathetic nervous activity (SNA) (Julien, 2006). Oscillations at this frequency have also been observed in cerebral haemodynamics measured with fNIRS (Obrig et al., 2000; Yucel et al., 2016) and intraoperative multispectral optical intrinsic signal imaging (Rayshubskiy et al., 2014). However, the origins of such signals are unclear, and separating the effects of ABP fluctuations from vasomotion (which is usually regarded as distinct) on cerebral haemodynamics is an open challenge.

Nevertheless, the TCD literature provides compelling reason to believe that ABP fluctuations should contribute to the BOLD fMRI signal. Measurement of the coupling between fluctuations in ABP and CBFV in intracranial arteries has found widespread use as a clinically useful means of assessing CA (Zhang et al., 1998; Panerai et al., 2002; Panerai, 2008), and nonlinear modeling estimates that ABP accounts for 60% of the predictive power of CBFV fluctuations in the MCA (Mitsis et al., 2004). Although TCD has been widely used to assess both CVR and CA in research and clinical practice (Willie et al., 2011), more recently fMRI has emerged as a powerful tool for mapping CVR across the brain (Pillai and Mikulis, 2015), and the feasibility of obtaining BOLD fMRI based measures of CVR from spontaneous CO_2_ fluctuations has also been demonstrated (Golestani et al., 2016). So far, these advances in measuring cerebrovascular function with fMRI have not yet extended into the domain of CA. However, the TCD literature proves that blood pressure related spontaneous CBFV fluctuations provide an effective means of characterizing CA, which is promising for the development of an equivalent whole-brain fMRI method.

In this study we explore the relationship between systemic fluctuations in blood pressure and the resting-state fMRI signal. We measure beat-to-beat blood pressure fluctuations concurrently with single-echo fMRI at 3T and multi-echo fMRI at 7T, and show that widespread patterns of correlations exist in low frequency BOLD signals, which we posit are due to fluctuations in CBF associated with CA.

## Materials and Methods

### Experimental Protocol

#### Magnetic Resonance Imaging Acquisition

The study consisted of two separate experiments conducted on two different scanners. Nine healthy volunteers (age 22–37 years) were recruited for a 3T session to collect single-echo fMRI data (3T) and five additional healthy volunteers (age 30–41 years) were recruited for a 7T session to collect multi-echo fMRI data (7T-ME). All participants gave written informed consent, and the School of Psychology Cardiff University Ethics Committee approved the study in accordance with the guidelines stated in the Cardiff University Research Framework (version 4.0, 2010).

The 7T scan protocol was added to enable us to address the origin of MAP correlated fMRI signal changes. Primarily this was achieved by using a multi-echo acquisition, which allows us to separate the pure BOLD component from the fMRI signal. Furthermore, the session consisted of two scans with differing acquisition parameters (see section “Multi-Echo Fit” below for theoretical details).

##### 3T

Two twenty-minute rs-fMRI runs were acquired on a 3T GE HDx scanner (GE Healthcare, Milwaukee, WI, United States) with an eight-channel receive head-coil using a gradient-echo EPI readout with a single echo time (TR = 2000 ms; TE = 35 ms; flip angle (α) = 90°; FOV = 224 mm; 3.5 mm^2^ in-plane resolution; 33 slices (3.5 + 0.5 mm gap), SENSE (GE ASSET) acceleration factor = 2). Whole-brain T_1_-weighted anatomical images were acquired using an FSPGR sequence (FOV = 256 mm, TR = 7900 ms, TE = 3 ms, 172 contiguous sagittal slices, 1 mm^3^ isotropic).

##### 7T-ME

Two ten-minute eyes-closed rs-fMRI runs were acquired on a 7T Siemens MAGNETOM scanner (Siemens Healthcare GmbH, Erlangen, Germany) equipped with a single-channel transmit/32-channel receive head coil (Nova Medical, Wilmington, MA, United States). The CMRR SMS-EPI sequence (R015) (Moeller et al., 2010) was used to acquire multi-echo multiband EPI data with three echoes using the following parameters: Scan 1 – [TR = 1000 ms; TE_1/2/3_ = 8.14/21.47/34.8 ms; flip angle (α) = 35°; FOV = 220 mm; 2.4 mm^2^ in-plane resolution; 36 slices (2.5 mm thick); multiband factor = 4; GRAPPA acceleration factor = 2] and Scan 2 – [TR = 500 ms; TE_1/2/3_ = 8.14/21.47/34.8 ms; flip angle (α) = 90°; FOV = 220 mm; 2.4 mm^2^ in-plane resolution; six slices (2.5 mm thick); multiband factor = 1; GRAPPA acceleration factor = 2]. Whole brain T_1_-weighted anatomical images were acquired using an MPRAGE sequence (FOV = 220 mm, TR = 2200 ms, TE = 3 ms, TI = 1050 ms, 224 contiguous sagittal slices, 0.7 mm^3^ isotropic).

#### Physiological Monitoring

Concurrent physiological traces were recorded for all runs and sampled at 500 Hz (CED, Cambridge, United Kingdom). This included using photoplethysmography (PPG) to measure pulse waveforms for deriving cardiac information, a pneumatic respiratory belt for timing and relative respiration volume measures, capnography for measuring expired partial pressure of end-tidal carbon dioxide (P_ET_CO_2_). The CareTaker system (Biopac) was used to measure beat-to-beat blood pressure with a cuff attached to the first digit of the hand (thumb). The system uses the cuff to pneumatically sensor the arterial pressure wave, and estimates beat-to-beat systolic and diastolic blood pressure via analysis of the timing between different components of the pulse waveform (Baruch et al., 2011), and has been validated against gold standard arterial line measurements (Baruch et al., 2014).

### Data Analysis

#### Preprocessing

Data were preprocessed and registered to a standard space using a pipeline created with AFNI, FSL, and in-house code. Preprocessing of 3T and 7T-ME data consisted of the same following steps: (1) De-spiking; (2) Motion correction by registering all volumes to the first one. For 7T-ME scans steps 1–2 were performed separately for each echo time dataset, then a nonlinear fit was performed to create S_0_ and R_2_^∗^ datasets (see section “Multi-Echo Fit” below). Subsequent steps were performed separately for S_0_ and R_2_^∗^ datasets. (3) Nuisance regression with pre-whitening (Bright et al., 2017) to remove cardiac and respiratory related noise (Glover et al., 2000; Birn et al., 2008; Chang et al., 2009), end-tidal CO_2_ fluctuations (convolved with HRF), and six estimated motion parameters; (4) Slice time correction; (5) Non-linear registration to 2 mm MNI space; (6) De-trending and motion censoring in a single step, with the top 5% of volumes most severely corrupted by motion (according to framewise displacement) being censored. Censored time points were replaced with interpolated values calculated from neighboring (non-censored) time points (NTRP option in 3dTproject) in order to keep to the data temporally consistent for subsequent wavelet decomposition. A discrete wavelet transform was then performed on the preprocessed data (see section “Maximum Overlap Discrete Wavelet Transform” below). Note that physiological noise correction was performed on unfiltered data.

For each subject gray matter (GM) masks were created from segmented T_1_ images, with GM voxels defined as those with a partial volume estimate greater than 66%. GM masks were used in subsequent parts of the analysis, and GM mask averaged time series were calculated for 3T and 7T-ME data for estimating the global lag with blood pressure (see section “Blood Pressure Correlation” below).

#### Multi-Echo Fit

Assuming a mono-exponential decay, the signal across multiple echo times can be described according to Eq. 1.

(1) $$S(\mathrm{TE})=S_{0}e^{-R_{2}^{*}\mathrm{TE}}$$

Where S_0_ is spin density weighted signal intensity at zero echo time and R_2_^∗^ is the apparent transverse relaxation rate (inverse of relaxation time T_2_^∗^). S_0_ is modulated by changes in apparent T_1_ (e.g., due to inflow) and bulk motion and related spin history effects, whereas R_2_^∗^ reflects magnetic field homogeneity and thus, due to blood oxygenation induced changes in microscopic susceptibility, the source of the BOLD effect (Buxton, 2013). NVC related functional responses in gradient-echo fMRI are considered to be driven almost entirely by R_2_^∗^ changes, which has motivated the use of multi-echo acquisitions for separating neuronal from non-neuronal signal components (Posse, 2012; Kundu et al., 2017), based on the rationale that non-neuronal components (i.e., not flow related) are mostly restricted to changes in S_0_. It should be noted that inflow effects on S_0_ are driven by changes in flow velocity through an imaging slice, and so may partially reflect changes in CBF, however, this effect is generally considered to be small in multi-slice acquisitions with standard TRs (Gao and Liu, 2012).

However, as discussed above, there are non-neuronal contributors to flow, whose activity in principle should manifest primarily in the form of R_2_^∗^ changes. Ignoring the effects of macroscopic field inhomogeneity, more generally R_2_^∗^ changes are driven by CBF dynamics (via the changes in blood oxygenation they produce), whether they are neuronally driven or not. The motivation for collecting 7T-ME data as part of this study is that it allows us to determine to what extent MAP-fMRI correlations are driven by changes in R_2_^∗^ and S_0_, and thus whether or not they are related to changes in CBF. Moreover, as S_0_ is determined by the steady-state longitudinal magnetization it is intrinsically dependent on acquisition parameters like TR and flip angle. Thus, any significant S_0_ fluctuations are likely to be modulated between 7T-ME scans 1 and 2, whereas R_2_^∗^ fluctuations will not.

For each voxel and TR, 7T-ME data were fit to the mono-exponential signal model with a nonlinear least-squares approach using the Levenberg-Marquardt algorithm (Galassi, 2009), creating S_0_ and R_2_^∗^ datasets. In multi-echo fMRI studies numerous physical limitations restrict the number of echo times that can be achieved to a small number, in this case three, which presents a challenge for accurate estimation of relaxation rates (Gowland and Bowtell, 2007), and sample-by-sample parameter estimates are considered to be noisy (Kundu et al., 2012). Compared with previous studies, here we benefit from the higher SNR afforded by 7T to improve sample-by-sample parameter estimates, and we performed simulations to better understand the precision with which R_2_^∗^ and S_0_ can be measured using our nonlinear fit approach (details included in Supplementary Material). These simulations demonstrate that across the expected range of R_2_^∗^ values, our choice of echo times allows us to estimate both R_2_^∗^ and S_0_ parameters without bias.

#### Maximum Overlap Discrete Wavelet Transform

Wavelet transforms provide a way of decomposing the total variance within a time series into different frequency scales [see Bullmore et al. (2004) for an fMRI focused review]. They are conceptually similar to Fourier transforms, but because wavelets are compactly supported (i.e., transient, not extending infinitely like sine waves), they provide sensitivity to non-stationary features within the scales of the decomposition. Thus, wavelet coefficients (WC) provide a “time-frequency” representation of data, including both temporal and spectral information, analogous to moving-window Fourier transforms. This makes the discrete wavelet transform useful for analyzing real-world physiological data, which are expected to be non-stationary, and the multi-resolution analysis allows signal energy to be decomposed into distinct frequency bands. Given that fluctuations in MAP are expected to show more power in certain frequency bands, likely reflecting different underlying mechanisms, in this study we used a wavelet transform to identify the frequency scale of interest for the relationship between MAP and fMRI signals.

For each voxel time series the maximum overlap discrete wavelet transform (MODWT) was used to decompose the signal into six scales (Witcher, 2015). The MODWT used a fourth-order Daubechies wavelet filter as has been used previously for fMRI applications (Bullmore et al., 2001; Patel and Bullmore, 2016). The central frequency (*f_c_*) and band between lowest (*f_low_*) and highest (*f_high_*) frequencies contained within each scale (*j*) depends on the sampling rate (*TR*), and is given by Eqs 2 and 3.

(2) $$f_{\text{low}}-f_{\text{high}}=\frac{1}{2^{\left(j+1\right)}\mathrm{TR}}-\frac{1}{2^{j}\mathrm{TR}}$$

(3) $$f_{\text{c}}=\frac{f_{\text{low}}+f_{\text{high}}}{2}$$

In summary, each fMRI time series consisting of N volumes was decomposed into six frequency scales, each composed of N WCs. For reference the scale frequency bands for the TRs used in this study are given in Table 1 and the frequency response of the filters at each scale are shown in Supplementary Figure 1.

**Table 1:** Table showing the frequency range (Hz) for each scale of the MODWT.

Scale	TR (s)		
	2	1	0.5
1	0.125–0.250	0.250–0.500	0.500–1.000
2	0.063–0.125	0.125–0.250	0.250–0.500
3	0.031–0.063	0.063–0.125	0.125–0.250
4	0.016–0.031	0.031–0.063	0.063–0.125
5	0.008–0.016	0.016–0.031	0.031–0.063
6	0.004–0.008	0.008–0.016	0.016–0.031

#### Blood Pressure Correlation

Beat-to-beat systolic (SBP) and diastolic (DBP) blood pressure time series were processed in-house with a robust outlier removal procedure and resampled to the relevant TR. Mean arterial pressure (MAP) was estimated according to Eq. 4 (Brzezinski, 1990).

(4) $$\mathrm{MAP}=\frac{2}{3}(\mathrm{DBP})+\frac{1}{3}(\mathrm{SBP})$$

**FIGURE 1:**
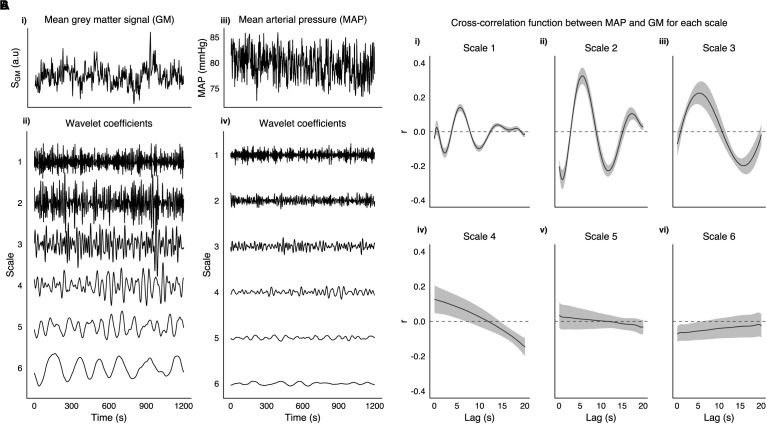
**A** (i) Shows the mean gray matter signal from 3TSE data in a representative subject along with corresponding mean arterial pressure trace (iii). Wavelet coefficients at 6 scales are shown for both GM (ii) and MAP (iv). **(B)** Group mean cross-correlation function between MAP and GM for each scale (i–vi), with shaded area representing SEM.

MAP time series were decomposed with a MODWT into the same six frequency scales as the fMRI data. Figure 1 shows a MAP time series and its WCs for a representative subject. For each run the cross-correlation between the average gray matter signal and temporally displaced MAP signals was calculated. The raw cross-correlation functions were then fit to a set of Legendre polynomial functions of increasing order (until R^2^ > 0.95, or up to a maximum order of 10) to obtain a smooth function from which the lag between MAP and fMRI fluctuations was obtained. Voxelwise correlations between MAP and scale matched fMRI WCs for all frequency scales were calculated with the globally optimized lag. Additionally, although more susceptible to noise, we calculated a voxelwise lag using the same procedure and then calculated corresponding correlations between MAP and scale matched fMRI WCs.

It should be noted that in contrast with the discrete wavelet transform (DWT), which is orthogonal (like the discrete Fourier transform), the MODWT is an oversampled transform in which there is some redundancy (i.e., coefficients are not completely independent). This means that the effective degrees of freedom are reduced, which impacts on statistical parameters. Additionally, the practice of filtering time series and allowing temporal shifts changes the null distribution of Pearson’s correlations (Bright et al., 2017). To address these potential confounds on statistical inference we performed a permutation analysis to determine the correct null hypothesis. The approach, which has been used previously (Bright et al., 2017), estimates the null distribution with phase-randomized versions of the MAP time series. For each subject 1000 phase-randomized MAP traces were correlated with fMRI to get a voxel-wise estimate of the correlation null distribution from which *p*-values were calculated.

#### Group Level Analysis

For each frequency scale, subject level MAP - fMRI WC correlation maps were entered into an independent two-tailed *t*-test, from which group-level correlation and *Z*-score maps were derived. The mean GM correlation and *Z*-score values were calculated as a means of identifying the scale with the strongest MAP vs. fMRI coupling. Subsequent analyses of 7T-ME data are restricted to the scale with the matched frequency. Test–retest repeatability was assessed using spatial correlation between Scan 1 and Scan 2 for 3T. For 7T-ME data, Scan 2 parameters were chosen differently from Scan 1 from a theoretical perspective to maximize any potential inflow effect in the S_0_ signal (Gao and Liu, 2012). For the different sources of image contrast, R_2_^∗^ and S_0_, to assess their relative contributions to MAP – fMRI correlations, the absolute value of average GM vs. MAP correlations were entered into a two way repeated measures ANOVA.

Note, that although a wavelet transform was used to decompose MAP and fMRI signals into different frequency scales, we also looked at the effect of MAP on unfiltered data by regressing unfiltered optimal lag MAP traces onto voxel-wise unfiltered fMRI data (see Supplementary Figure 4).

## Results

### 3T

#### Global Lag

Figure 1A, shows an example taken from a representative subject of GM and MAP traces, and their respective WCs at the six frequency scales listed in Table 1. Figure 1B shows the group mean cross-correlation functions between MAP and GM WC time series for each scale of the MODWT. Scales 1–3 all show clear maxima with a similar degree of lag (5.75, 5.50, and 5.25 s for scales 1–3, respectively).

#### MAP vs. fMRI Correlation

**FIGURE 2:**
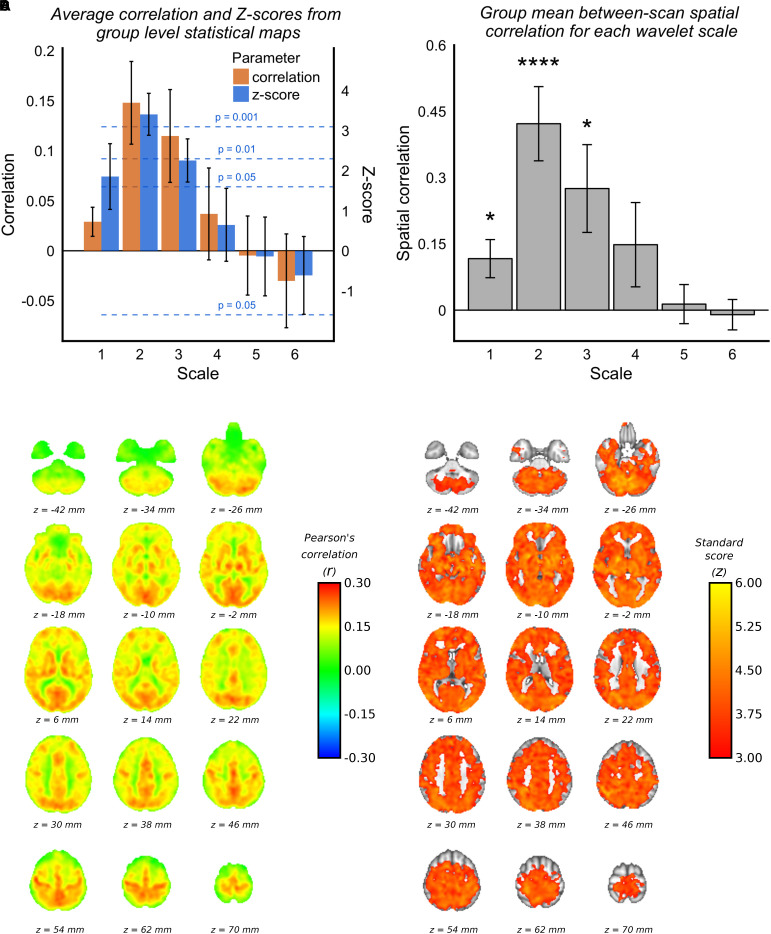
3TSE data. **(A)** Mean correlations and *Z*-scores (across voxels) from group mean statistical parameter maps for each scale. Dotted lines indicated the *p*-value for give *z*-score vales. **(B)** Spatial correlation scans 1 and 2 MAP-fMRI correlation maps for each scale (^∗^*p* < 0.05, ^∗∗^*p* < 0.01, ^∗∗∗^*p* < 0.001, and ^∗∗∗∗^*p* < 0.0001). **(C)** Group mean MAP – fMRI correlation map for scale 2 WC and corresponding *Z*-scores. **(D)** Correlations are those at the optimal lag values as shown in Figure 1.

Voxelwise group average correlations (at optimal lag) are shown for scale 2 WCs only in Figure 2C, along with corresponding *Z*-scores in Figure 2D. Figure 2A shows the mean correlation values and *Z*-scores within the voxelwise group maps for all scales (at optimal lag), and it can be seen the scale 2 has the highest mean correlation value and highest mean *Z*-score value. It can be seen by the standard deviation error bars that the majority of voxels at scale 2 have a *Z*-score >3.1 (*p* = 0.001). Figure 2B demonstrates that the spatial correlation between scans (i.e., within subject agreement) is also highest for scale 2 compared with the other scales. As documented in Table 1, the frequency band for scale 2 for 3TSE data (TR = 2s) is 0.063–0.125 Hz, which corresponds to a central frequency () of ∼0.1 Hz.

As stated in Section “Maximum Overlap Discrete Wavelet Transform”, permutation tests based on phase-randomized MAP time series were used to estimate the correlation null distribution for each subject on a voxel-wise basis. Supplementary Figure 3A shows the correlation null distributions estimated for each subject, and the mean GM correlation value. For each subject the null distribution is non-zero, as expected due to the effect of temporal shifting, but in every case the true mean GM correlation with MAP is more than three standard deviations removed from the null correlation. Supplementary Figure 2 shows subject level correlation maps along with associated threshold permutation test *p*-values.

For reference, Supplementary Figure 4 shows the estimated effect size of unfiltered MAP fluctuations on fMRI data. The average GM effect size across subjects (±SD) is 0.01% BOLD/mm Hg (±0.006). Across subjects the average absolute maximum deviation in MAP is ∼12 mm Hg, which suggests fairly modest total BOLD signal changes on the order of ∼0.12% are expected, but given the heterogeneity evident in Supplementary Figure 4, it is clear that in some regions this may be as large as ∼0.5%.

#### Voxelwise Lag

**FIGURE 3:**
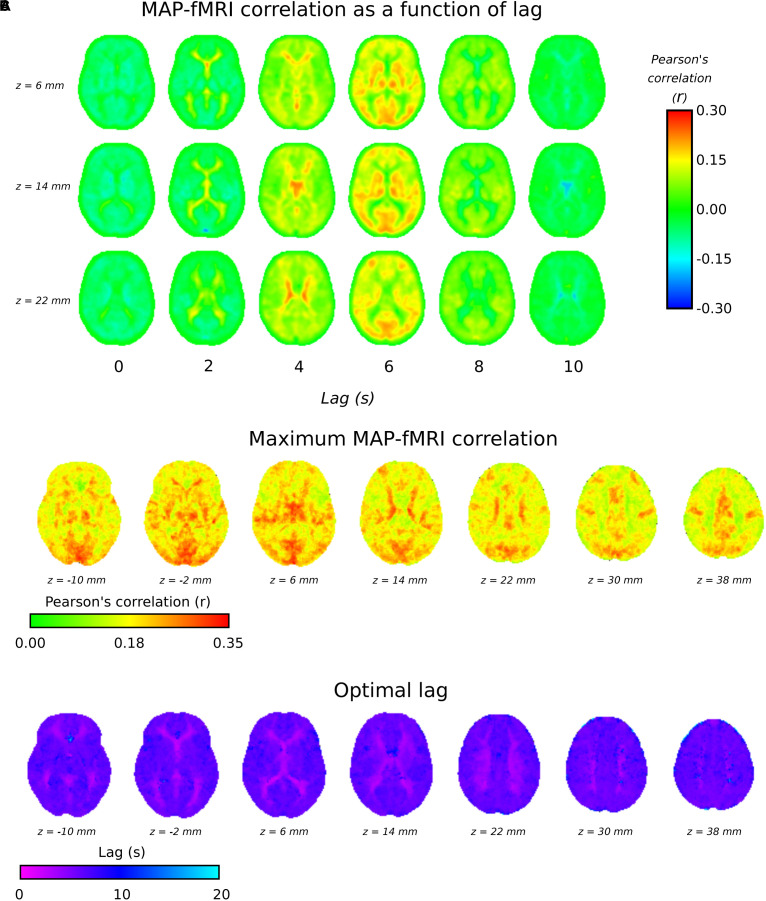
3TSE data. **(A)** MAP – fMRI as a function of lag with respect to MAP, in 2 s intervals. **(B)** The maximum correlation (i.e., arg max of cross-correlation function) and the associated lag time **(C)**.

Figure 3A shows group level MAP – fMRI correlations as a function of lag with respect to MAP. It shows how a spatially structured pattern of fMRI signal changes evolves over time in response to MAP fluctuations. Figure 3B,C shows the maximum correlation and the lag at which it is seen, respectively. The lag time in cortical gray matter appears relatively uniformly distributed at ∼5 s, in good agreement with the GM signal global lags shown in Figure 1. Interestingly, there is a correlation pattern that emerges earlier (∼2–4 s), which appears in deep white matter structures and in the areas bordering the lateral ventricles. Figure 3B shows that there are widespread correlations with MAP, albeit with different lags, extending across the whole brain.

### 7T

**FIGURE 4:**
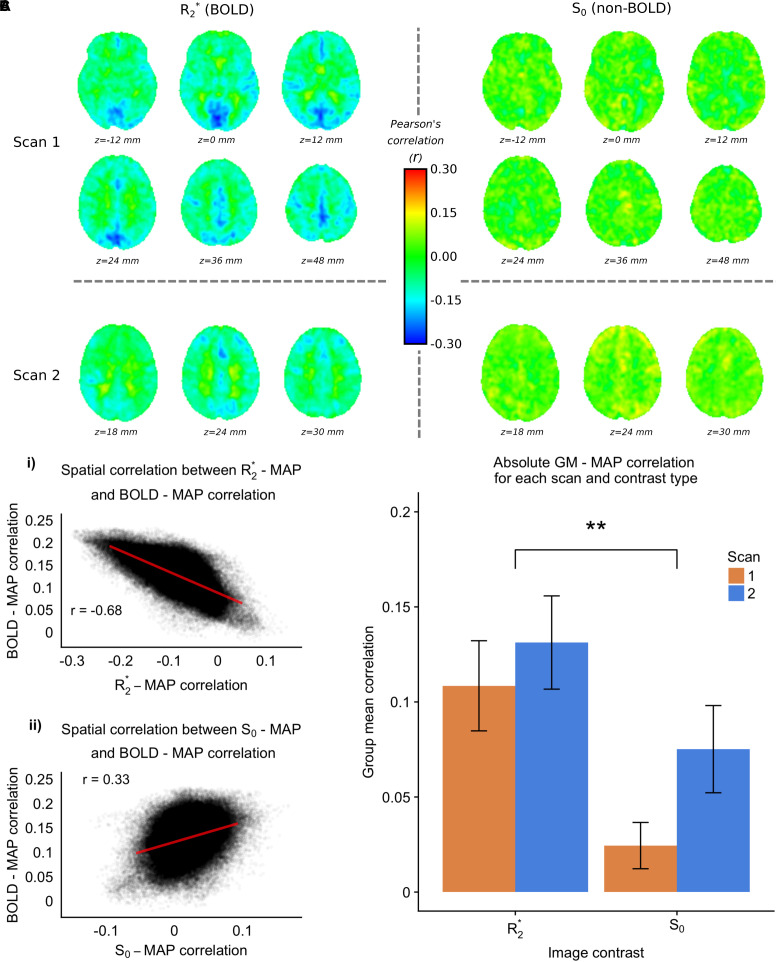
7TME data. **(A)** group level MAP – fMRI correlation maps for R_2_^∗^ and S_0_ and scans 1 and 2, at the frequency scale corresponding to 0.063 – 0.125 Hz (scales 3 and 4 for scans 1 and 2 respectively). **(B)** (i) Spatial correlation between 7TME MAP – R_2_^∗^ correlation maps, and 3TSE MAP – fMRI correlation maps. (ii) Spatial correlation between 7TME MAP – S_0_ correlation maps, and 3TSE MAP – fMRI correlation maps. **(C)** Bar chart showing group mean GM correlations (absolute value) for R_2_^∗^/S_0_ and scans 1 and 2 (^∗^*p* < 0.05, ^∗∗^*p* < 0.01).

The 7T-ME data allows us to tease apart the different sources of contrast underlying the MAP – fMRI correlations. Figure 4A shows group level voxelwise MAP – R_2_^∗^ and MAP – S_0_ correlations for Scan 1 and Scan 2. R_2_^∗^ – MAP correlations show a similar pattern to 3T fMRI – MAP correlations, with well defined gray/white matter contrast and matching areas of high magnitude correlations (e.g., in occipital cortex). Note that negative R_2_^∗^ – MAP correlations are equivalent to positive MAP – fMRI (3T) correlations, as a decrease in R_2_^∗^ corresponds to a lengthening of T_2_^∗^ and a positive increase in BOLD signal. Figure 4B shows the spatial correlations between R_2_^∗^ – MAP and S_0_ – MAP correlation maps, and 3T BOLD – MAP correlation maps. Compared with S_0_ – MAP correlations, R_2_^∗^ – MAP correlation maps are more spatially similar to 3T BOLD – MAP correlation maps, with a Pearson’s correlation of −0.68 vs. 0.33 of S_0_ – MAP, which amounts to ∼4 times as much variance explained. Note that the negative correlation in Figure 4Bi is due to the inverse relationship between R_2_^∗^ and the BOLD signal (a BOLD signal increase results from less dephasing, i.e., a decrease in relaxation rate).

Figure 4C shows the group mean GM absolute correlation values for R_2_^∗^ and S_0_ scans 1 and 2. A two-way repeated measures ANOVA revealed a significant effect of contrast (R_2_^∗^ > S_0_), but no effect of scan. Following the rationale outlined in Section “Multi-Echo Fit,” this would suggest that the MAP correlated fMRI signal has a BOLD origin related to changes in CBF. Furthermore, there appears to be minimal non-BOLD contribution, as scan number did not significantly modulate the MAP-S_0_ correlation values.

## Discussion

### Blood Pressure Correlation

To our knowledge, this study is first to demonstrate that MAP LFOs are positively correlated with fMRI LFOs within the frequency band between 0.063 and 0.125 Hz. These correlations appear highly spatially structured, with strong gray/white matter contrast, and are repeatable between subjects with a spatial correlation of ∼0.42. Results from the 7T-ME data suggest that fluctuations in MAP lead to gray matter signal fluctuations in BOLD fMRI that are primarily related to CBF, given that they are related to changes in R_2_^∗^ and relatively independent of acquisition parameters. This is consistent with a large TCD literature that shows beat-to-beat fluctuations in blood pressure result in measurable changes in CBFV in large intracranial arteries (Aaslid et al., 1989; Diehl et al., 1991; Blaber et al., 1997; Kuo et al., 1998; Zhang et al., 1998), lagged by ∼2 s, with MAP preceding cerebral blood flow velocity (CBFV). As BOLD fMRI is sensitive to deoxygenated blood volume compartments (i.e., capillary and venous) that are downstream of large intracranial arteries that are insonated with TCD, one might assume an extended delay that would allow changes to propagate along the vasculature tree. Given the obvious logic of this, the fact that the true results show that fMRI precedes MAP by ∼5.5 s most likely reflects differences in how MAP is measured in this study compared with previous reports. Continuous non-invasive MAP measurement ismost often done with the Finapres system. However, as this is not MRI compatible we instead used the Caretaker system, from which beat-to-beat blood pressure is estimated from an analysis of the pulse wave in the periphery. Although the Caretaker is validated against invasive arterial line measurement (Baruch et al., 2014), and shows good agreement, this study does not include any investigation of timing differences. However, as it is based on Pulsewave Decomposition Analysis (PDA) of the peripheral arterial pressure wave, transit time differences must be considered.

Instantaneous blood pressure is an idealized concept, as in reality local changes in pressure take time to propagate along the vascular tree, which depends on stiffness of the different arterial vascular beds (Chen et al., 2009). As such, all blood pressure measurements are temporally shifted surrogates of the true aortic value, by which MAP is usually defined. Beat-to-beat blood pressure is predominantly regulated in response to the activity of baroreceptors, which are located in the aortic arch and carotid sinus. Thus, pressure changes are detected centrally, which leads to systemic changes in the downstream vasculature in response. The self-evident logic of cerebral autoregulation is that cerebral hemodynamics change in response to fluctuations MAP. Thus, although we have observed fMRI signals that precede MAP signals, it seems very unlikely that this is a causative effect. It is more likely that the lag, in which fMRI precedes Caretaker MAP, can be explained by systemic vascular transit time differences. Furthermore, this suggests one should be cautious about interpreting lags between cerebral and peripheral hemodynamics, as they likely depend on the complex interaction of multiple factors, including stiffness of the different arterial vascular beds, and the interplay between autonomic and myogenic activity. Furthermore, the lag time would be expected to account for the fact the flow changes will take time to propagate along the vascular tree. Delayed fMRI responses to hypercapnia challenges are frequently observed on the order of 8–15 s (Blockley et al., 2011; Murphy et al., 2011), although potentially longer in patient groups (Duffin et al., 2015; Donahue et al., 2016), and are presumed to contain both gas bolus transit time and vascular reactivity information. However, untangling the different factors that influence these timing differences could present an interesting new avenue of research. Central (aortic) arterial stiffness is likely to contribute greatly to the measured lag, and so experiments that can separate these general systemic effects from more specific cerebral vascular ones are desirable, and there are novel MRI methods for quantifying aortic stiffness would allow for this to be done within the same imaging session (Fielden et al., 2008; Grotenhuis et al., 2009; Langham et al., 2011). The voxelwise lag analysis shows that lag times for white matter are shorter than gray matter. Considering the correct directionality of the lag structure, this perhaps make sense, as it suggests that fluctuations in gray matter are followed by fluctuations in white matter, and finally by fluctuations in peripheral MAP measurements. Thus, this is consistent with the fMRI literature showing low frequency fMRI signals of systemic origin that are delayed in white matter, as blood arrival time is extended with respect to gray matter (van Gelderen et al., 2008).

From these data we cannot know the exact origin of these BP correlated fMRI fluctuations. Previous fMRI studies have found signal fluctuations that are correlated with peripheral measures of vascular tone, such as NIRS in fingers/toes (Tong and Frederick, 2010; Tong et al., 2012, 2013) or amplitude of photoplethysmography (PPG) (van Houdt et al., 2010; Ozbay et al., 2018). These observations support the existence of endogenous systemic LFOs, which propagate throughout the entire cardiovascular system, appearing as synchronized, but out of phase, oscillations at different vascular sites. A common systemic source is one explanation for the MAP correlated fMRI signals we have measured, and a potential candidate for this systemic origin is SNA. The MODWT reveals that MAP coupled fMRI LFOs are strongest in the frequency band centered at ∼0.1 Hz, the frequency of Mayer waves, which are defined in terms of their coherence with SNA (Julien, 2006). SNA regulates blood pressure via modulation of peripheral vascular tone (Fisher and Paton, 2012), but also potentially influences cerebrovascular tone (Brassard et al., 2017), and increases in SNA elicited by post exercise induced ischemia have been shown to decrease compliance in the brain’s major arteries (Warnert et al., 2016). Orthostatic challenges such as lower body negative pressure (LBNP), which are associated with increases in SNA, lead to considerable reductions in MCA CBFV (Levine et al., 1994; Serrador et al., 2000; Zhang and Levine, 2007), and reductions in blood volume indicative of vasoconstriction in the brain’s largest arteries (Whittaker et al., 2017). Furthermore, studies have shown that ganglion blockade designed to dampen SNA, significantly alter the dynamics between MAP and CBFV (Zhang et al., 2002; Mitsis et al., 2009), suggesting autonomic neural control cerebrovascular tone likely plays a role in beat-to-beat CBF regulation.

### Cerebral Autoregulation

The time scale of the BP correlated LFO and its basis predominantly being changes in apparent transverse relaxation is strongly indicative of a CBF related cause. Compared with respiratory challenges for which the CBF response primarily probes CVR, the observed flow response associated with BP is likely to be related to the process of CA. Thus, whereas CVR is a measure of localized vascular integrity, i.e., the ability of arterial vessels to change their resistance, measures of CA relate to the systemic orchestrated vascular mechanisms that regulate CBF (Carrera et al., 2009). The LFO fluctuations we have observed in this study are correlated with BP measured in the periphery, and so are more related to CA than CVR. Impairments in CA associated with adverse cerebrovascular events such as ischaemic stroke and severe head injury have been well studied (Panerai, 2008), and are increasingly thought to play a role in the development of vascular dementia (Toth et al., 2017) and Alzheimer’s disease (Claassen and Zhang, 2011; den Abeelen et al., 2014). Despite the widespread clinical implications of pathological CA, its underlying mechanisms are still relatively poorly understood.

TCD is the most widely used modality for measuring CA, which despite having excellent temporal resolution and high suitability for clinical settings, is ultimately of limited value since the measurements are restricted to only the largest intracranial arteries. In contrast, fMRI has whole-brain sensitivity with millimeter resolution and so is a desirable tool for better understanding CA, and has the potential to deliver more predictive clinical measures. For example, CA is critical for keeping stable CBF in the penumbra region following ischaemic stroke (Xiong et al., 2017), so a method such as fMRI, which has the spatial resolution to resolve localized alterations, is promising as a more informative prognostic tool. In the TCD literature the transfer function between BP and CBFV is used to characterize CA, primarily through gain and phase shift. It is commonly assumed that a phase shift and low gain constitutes good CA (i.e., CBFV fluctuations are delayed with respect to BP and are dampened) (van Beek et al., 2008). In this study we observed a lag in fMRI with respect to MAP, which may be related to the phase shifts measured in TCD. Furthermore, although the effect-size of MAP on fMRI measured here appears small (Supplementary Figure 4), this may be due to the young healthy subject group. In patient groups with less effective CA both effect-size and lag may be modulated.

### Effect on Resting-State fMRI

This study provides the first step in characterizing the relationship between MAP and the fMRI signal, but further work is needed to address the degree to which MAP impacts functional connectivity measures. The estimated effect size on unfiltered data is relatively modest (∼0.01% BOLD/mm Hg), which across subjects on average amounts to total BOLD signal changes on the order of ∼0.1% across all gray matter, although total signal changes as large ∼0.5% are possible, depending on individual subject response variability. The results of the wavelet transformed data show that MAP fluctuations effect fMRI within a particular frequency band, and as in practice resting-state fMRI analyses never use raw unfiltered data, it is likely that the effect of MAP on functional connectivity metrics will depend on a variety of analysis choices, such as filter passband or window length in dynamic connectivity studies. These data also serve as a reminder that not all sources of BOLD contrast are neuronal in origin, and so multi-echo based approaches like ME-ICA (Kundu et al., 2012) are likely to be less effective. An interesting avenue of future research would be look at the effect of different echo combination and de-noising schemes to determine their impact.

## Conclusion

In this study we have shown that beat-to-beat fluctuations in BP are correlated with fluctuations in the resting-state fMRI that precede them by approximately 5.5 s, and which are strongest at the frequency band centered at ∼0.1 Hz. Using a multi-echo acquisition we were able to isolate the pure BOLD (R_2_^∗^) component of the BP correlated fMRI signal and have shown that it is the main source of contrast. This would indicate that it is changes in CBF that mediate this low frequency BP correlated signal, which we hypothesize is related to the process of CA. We propose that resting-state fMRI is a promising new tool for assessment of dynamic CA with high spatial resolution, which may prove to be a useful biomarker in a range of cerebrovascular and neurological conditions.

## Ethics Statement

The School of Psychology Cardiff University Ethics Committee approved the study in accordance with the guidelines stated in the Cardiff University Research Framework (version 4.0, 2010).

## Author Contributions

JW and KM conceived of the presented idea. JW, ID, MV, and MB collected the data. JW analyzed the data. All the authors provided critical feedback and helped shape the research, analysis, and manuscript.

## Conflict of Interest Statement

The authors declare that the research was conducted in the absence of any commercial or financial relationships that could be construed as a potential conflict of interest.
