# Corrigendum: Cerebral Autoregulation Evidenced by Synchronized Low Frequency Oscillations in Blood Pressure and Resting-State fMRI

**DOI:** 10.3389/fnins.2020.00544

**Published:** 2020-06-24

**Authors:** Joseph R. Whittaker, Ian D. Driver, Marcello Venzi, Molly G. Bright, Kevin Murphy

**Affiliations:** ^1^Cardiff University Brain Research Imaging Centre (CUBRIC), School of Physics and Astronomy, Cardiff University, Cardiff, United Kingdom; ^2^CUBRIC, School of Psychology, Cardiff University, Cardiff, United Kingdom; ^3^Department of Physical Therapy and Human Movement Sciences, Feinberg School of Medicine, Northwestern University, Chicago, IL, United States

**Keywords:** cerebral autoregulation, resting-state fMRI, blood pressure, cerebral physiology, LFO, BOLD, CBF

In the original article, there was an error. The article describes the lag structure between mean arterial pressure (MAP) and the fMRI signal, both globally and at a voxelwise level. A sign error contained within in-house analysis code led the authors to misattribute the directionality of this lag. In the article it is implied that MAP precedes fMRI by approximately 5.5 s, whereas in fact the fMRI signal precedes the MAP signal. There is no explicit error in the original text as it only states that there is a lag in fMRI “with respect to MAP,” which in itself is unfortunately not completely unambiguous, as the precise lag/lead terminology used in the literature is not standardized. However, the authors were still laboring under a misapprehension, and thus as a result a section of the Discussion section is misleading. This error does not alter the primary scientific conclusion of this article, which is that low frequency fluctuations in fMRI are associated with matched frequency fluctuations in MAP. However, the Discussion section includes a body of text which considers the relevance of this lag and posits some speculative physiological interpretations. As these discussion points are based on the aforementioned erroneous lag direction, they are no longer relevant to the reported results. Furthermore, there is an erroneous sentence in the Conclusion that states “fluctuations in the resting-state fMRI signal that are *delayed* by approximately 5.5 s,” as this use of the word delay implies MAP precedes fMRI. Finally, we correct the erroneous sentence in the Conclusion. The corrected paragraphs appear below.

The Discussion section, subsection Blood Pressure Correlations, paragraphs 1 and 2:

“To our knowledge, this study is first to demonstrate that MAP LFOs are positively correlated with fMRI LFOs within the frequency band between 0.063 and 0.125 Hz. These correlations appear highly spatially structured, with strong gray/white matter contrast, and are repeatable between subjects with a spatial correlation of ~0.42. Results from the 7T-ME data suggest that fluctuations in MAP lead to gray matter signal fluctuations in BOLD fMRI that are primarily related to CBF, given that they are related to changes in ${\text{R}}_{2}^{*}$ and relatively independent of acquisition parameters. This is consistent with a large TCD literature that shows beat-to-beat fluctuations in blood pressure result in measurable changes in CBFV in large intracranial arteries (Aaslid et al., 1989; Diehl et al., 1991; Blaber et al., 1997; Kuo et al., 1998; Zhang et al., 1998), lagged by ~2 s, with MAP preceding cerebral blood flow velocity (CBFV). As BOLD fMRI is sensitive to deoxygenated blood volume compartments (i.e., capillary and venous) that are downstream of large intracranial arteries that are insonated with TCD, one might assume an extended delay that would allow changes to propagate along the vasculature tree. Given the obvious logic of this, the fact that the true results show that fMRI precedes MAP by ~5.5 s most likely reflects differences in how MAP is measured in this study compared with previous reports. Continuous non-invasive MAP measurement is most often done with the Finapres system. However, as this is not MRI compatible we instead used the Caretaker system, from which beat-to-beat blood pressure is estimated from an analysis of the pulse wave in the periphery. Although the Caretaker is validated against invasive arterial line measurement (Baruch et al., 2014), and shows good agreement, this study does not include any investigation of timing differences. However, as it is based on Pulsewave Decomposition Analysis (PDA) of the peripheral arterial pressure wave, transit time differences must be considered.

Instantaneous blood pressure is an idealized concept, as in reality local changes in pressure take time to propagate along the vascular tree, which depends on stiffness of the different arterial vascular beds (Chen et al., 2009). As such, all blood pressure measurements are temporally shifted surrogates of the true aortic value, by which MAP is usually defined. Beat-to-beat blood pressure is predominantly regulated in response to the activity of baroreceptors, which are located in the aortic arch and carotid sinus. Thus, pressure changes are detected centrally, which leads to systemic changes in the downstream vasculature in response. The self-evident logic of cerebral autoregulation is that cerebral hemodynamics change in response to fluctuations MAP. Thus, although we have observed fMRI signals that precede MAP signals, it seems very unlikely that this is a causative effect. It is more likely that the lag, in which fMRI precedes Caretaker MAP, can be explained by systemic vascular transit time differences. Furthermore, this suggests one should be cautious about interpreting lags between cerebral and peripheral hemodynamics, as they likely depend on the complex interaction of multiple factors, including stiffness of the different arterial vascular beds, and the interplay between autonomic and myogenic activity. Furthermore, the lag time would be expected to account for the fact the flow changes will take time to propagate along the vascular tree. Delayed fMRI responses to hypercapnia challenges are frequently observed on the order of 8–15 s (Blockley et al., 2011; Murphy et al., 2011), although potentially longer in patient groups (Duffin et al., 2015; Donahue et al., 2016), and are presumed to contain both gas bolus transit time and vascular reactivity information. However, untangling the different factors that influence these timing differences could present an interesting new avenue of research. Central (aortic) arterial stiffness is likely to contribute greatly to the measured lag, and so experiments that can separate these general systemic effects from more specific cerebral vascular ones are desirable, and there are novel MRI methods for quantifying aortic stiffness would allow for this to be done within the same imaging session (Fielden et al., 2008; Grotenhuis et al., 2009; Langham et al., 2011). The voxelwise lag analysis shows that lag times for white matter are shorter than gray matter. Considering the correct directionality of the lag structure, this perhaps make sense, as it suggests that fluctuations in gray matter are followed by fluctuations in white matter, and finally by fluctuations in peripheral MAP measurements. Thus, this is consistent with the fMRI literature showing low frequency fMRI signals of systemic origin that are delayed in white matter, as blood arrival time is extended with respect to gray matter (van Gelderen et al., 2008).”

The Discussion section, subsection Cerebral Autoregulation, paragraph 2:

“TCD is the most widely used modality for measuring CA, which despite having excellent temporal resolution and high suitability for clinical settings, is ultimately of limited value since the measurements are restricted to only the largest intracranial arteries. In contrast, fMRI has whole-brain sensitivity with millimeter resolution and so is a desirable tool for better understanding CA, and has the potential to deliver more predictive clinical measures. For example, CA is critical for keeping stable CBF in the penumbra region following ischemic stroke (Xiong et al., 2017), so a method such as fMRI, which has the spatial resolution to resolve localized alterations, is promising as a more informative prognostic tool. In the TCD literature the transfer function between BP and CBFV is used to characterize CA, primarily through gain and phase shift. It is commonly assumed that a phase shift and low gain constitutes good cerebral autoregulation (i.e., CBFV fluctuations are delayed with respect to BP and are dampened) (van Beek et al., 2008). In this study we observed a lag in MAP with respect to fMRI, i.e., fMRI precedes MAP, which may be related to the phase shifts measured in TCD. Furthermore, although the effect-size of MAP on fMRI measured here appears small (Supplementary Figure 4), this may be due to the young healthy subject group. In patient groups with less effective CA both effect-size and lag may be modulated.”

The Conclusion:

“In this study we have shown that beat-to-beat fluctuations in BP are correlated with fluctuations in the resting-state fMRI that precede them by approximately 5.5 s, and which are strongest at the frequency band centered at ~0.1 Hz. Using a multi-echo acquisition we were able to isolate the pure BOLD (${\text{R}}_{2}^{*}$) component of the BP correlated fMRI signal and have shown that it is the main source of contrast. This would indicate that it is changes in CBF that mediate this low frequency BP correlated signal, which we hypothesize is related to the process of CA. We propose that resting-state fMRI is a promising new tool for assessment of dynamic CA with high spatial resolution, which may prove to be a useful biomarker in a range of cerebrovascular and neurological conditions.”

The authors apologize for this error and state that this does not change the scientific conclusions of the article in any way. The original article has been updated.
