# Supplementary figures and images for: Cerebral Autoregulation Evidenced by Synchronized Low Frequency Oscillations in Blood Pressure and Resting-State fMRI
Source: Front Neurosci. 2019 May 7;13:433. doi: 10.3389/fnins.2019.00433 (PMC6514145; doi:10.3389/fnins.2019.00433)

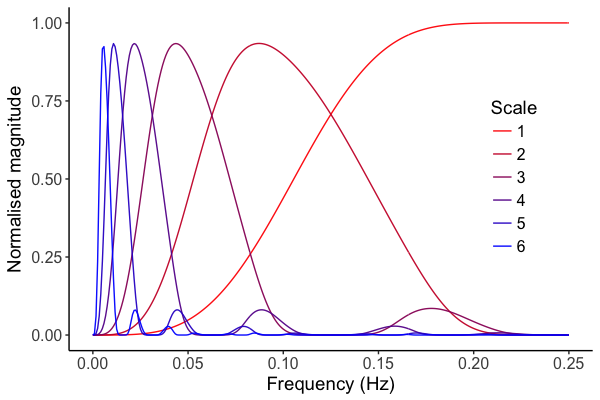

Supplement: Supplementary file 1 [file Image_1.TIFF]

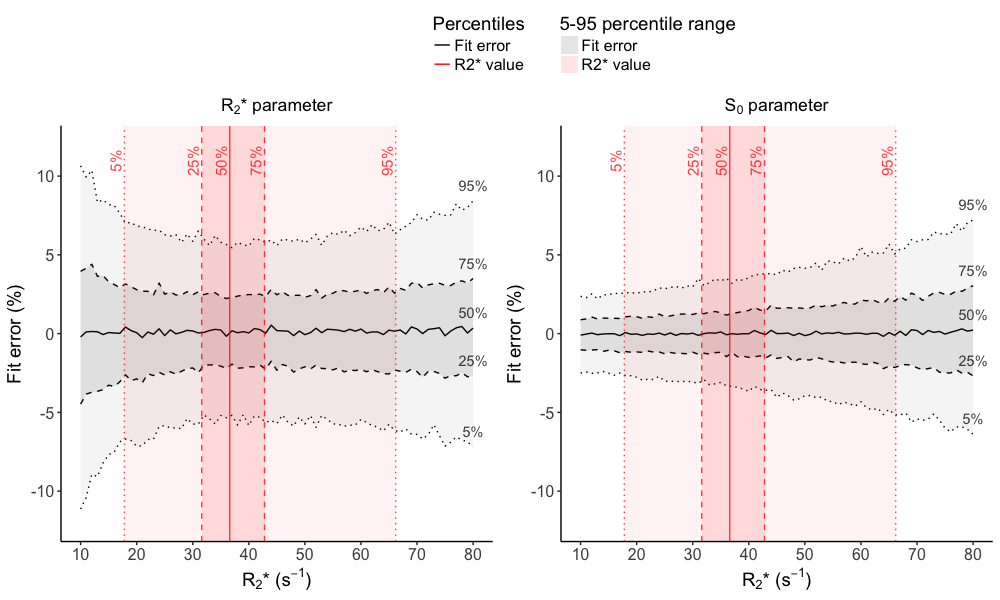

Supplement: Supplementary file 2 [file Image_2.TIFF]

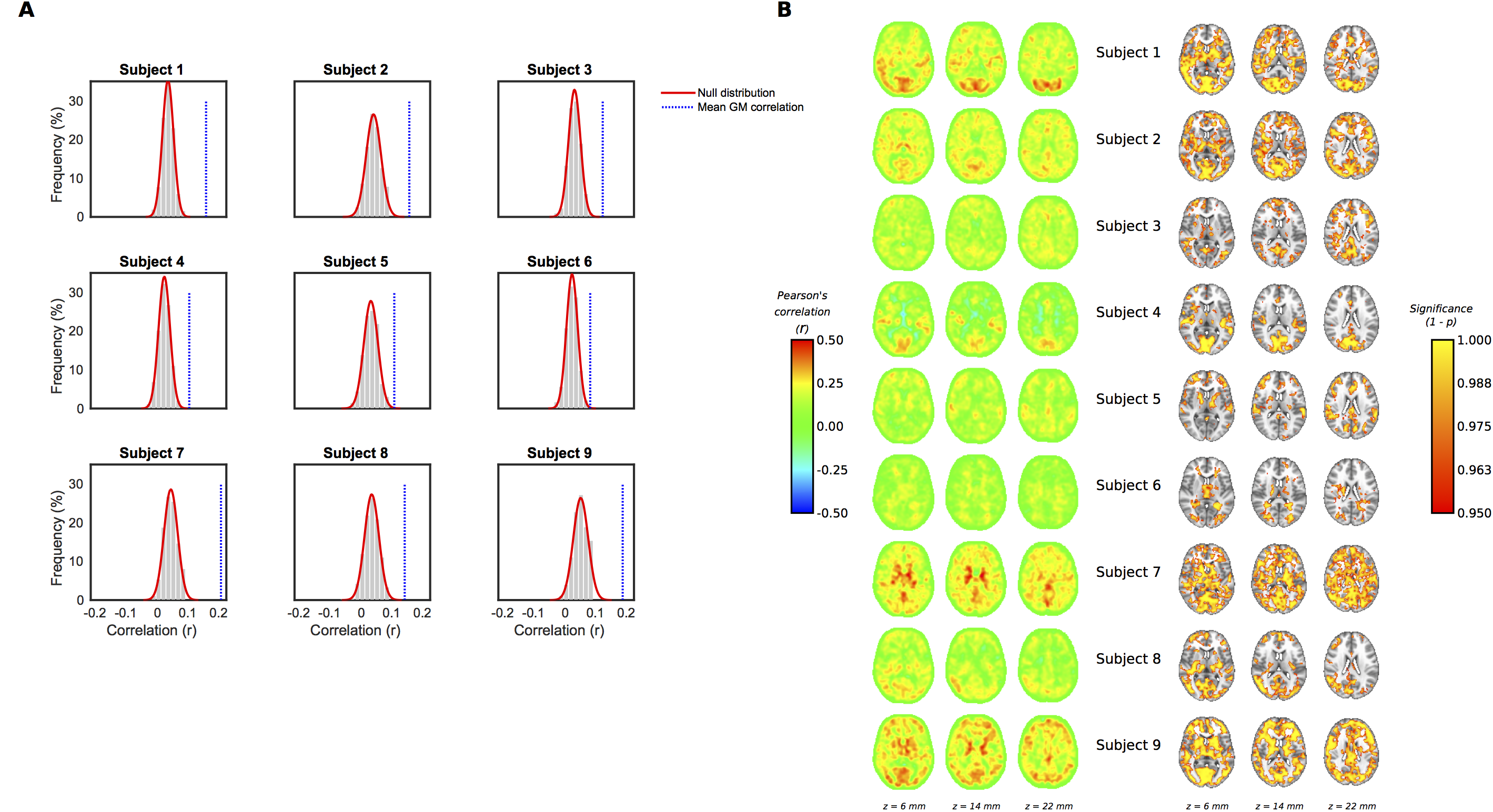

Supplement: Supplementary file 3 [file Image_3.TIFF]

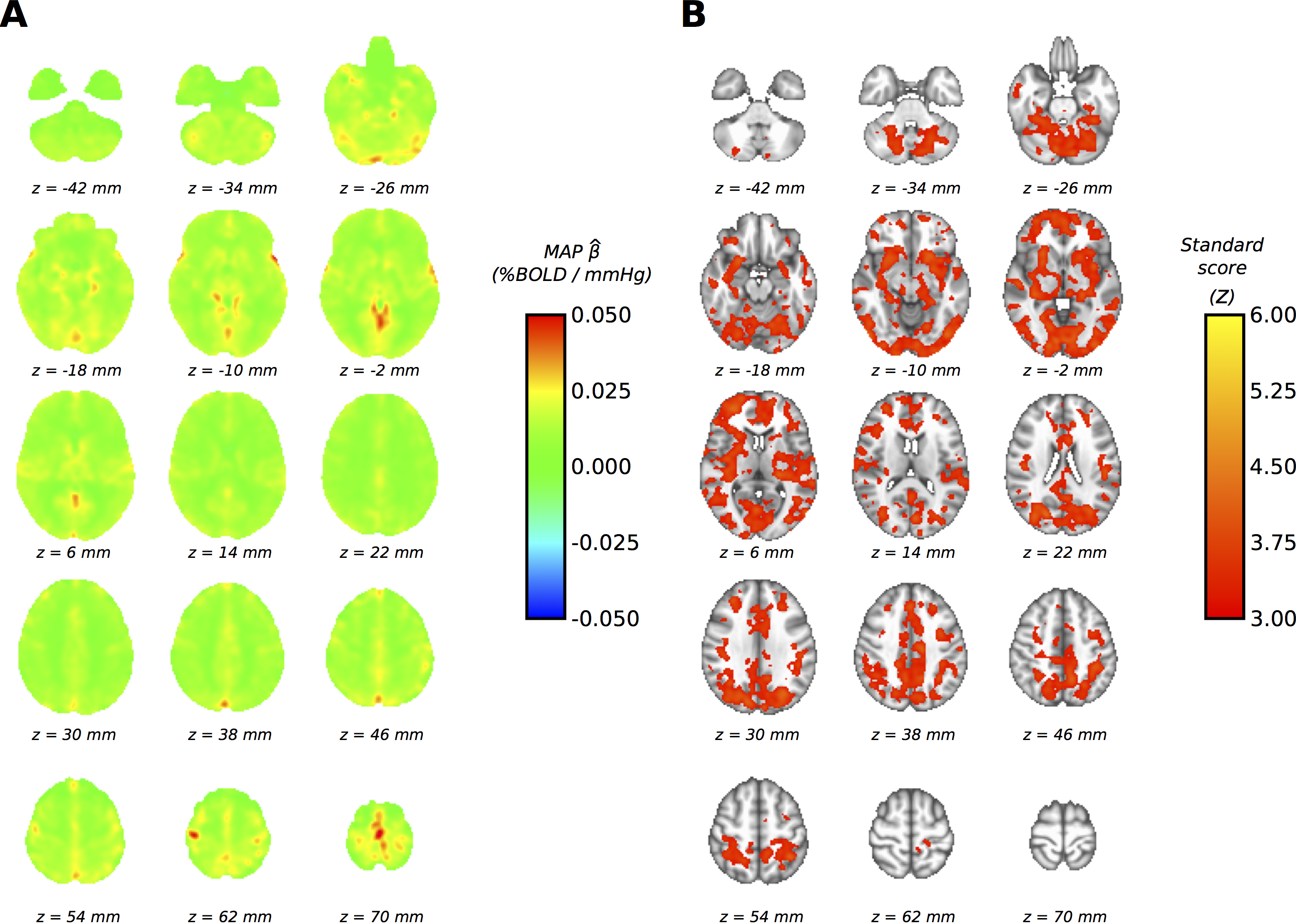

Supplement: Supplementary file 4 [file Image_4.TIFF]
