# Supplementary material for: Cerebral Autoregulation Evidenced by Synchronized Low Frequency Oscillations in Blood Pressure and Resting-State fMRI
Source: Front Neurosci. 2019 May 7;13:433. doi: 10.3389/fnins.2019.00433 (PMC6514145; doi:10.3389/fnins.2019.00433)
Supplement: Supplementary file 5 [file Table_1.DOCX]

# Supplementary material

## Wavelet Transform frequency response

<insert Supplementary Figure 1>

## Nonlinear exponential fit simulation

A simulation analysis was performed in order to assess the robustness of the nonlinear fit. 1000 exponential curves were simulated over a range of R_2_* values (10 – 80 s^-1^) with a fixed S_0_ value and Gaussian noise equivalent to a temporal SNR (tSNR) of 100. R_2_* values were selected within a physiologically plausible range, and are in agreement with the data, in which the 5^th^ and 95^th^ percentiles for subject 1 for grey matter are 18 s^-1^ and 66 s^-1^ respectively. The average grey matter tSNR in the same subject was calculated as the mean divided by the standard deviation of the echo 2 data, scaled by TE_2_ and a literature derived R_2_* value of 35 s^-1^, to obtain a S0 tSNR of 133, meaning the value of 100 used in the simulation was conservative. Supplementary Fig.2 shows the result of the simulations.

<insert Supplementary Figure 2>

Supplementary Fig. 2 shows that over the range of plausible R_2_* values and with our chosen TE values, the nonlinear fit does not produce any bias in parameter estimates. As expected the variability in R_2_* parameter estimates increases outside the range in which TE values are optimal for sampling the exponential decay. However, these simulation data demonstrate that our choice of echo times is close to optimal, as the R_2_* estimated parameter error variance is minimized within the robust range of R_2_* values. As expected S_0_ estimated parameter error variance is a function of R_2_* value, but over the interquartile range of R_2_* values, the interquartile range of parameter estimate error is ~ ±2.2% and ±1.3% for R_2_* and S_0_ respectively.

## Individual subject response and permutation tested p-values

<insert Supplementary Figure 3>

## Effect size in unfiltered data

<insert Supplementary Figure 4>

# Supplementary figure captions

Supplementary Figure 1: The frequency response for the different scales of the Maximum Overlap Discrete Wavelet using the Daubechies wavelet filter.

Supplementary Figure 2: Figure showing the percentage error in estimated R_2_* and S_0_ parameters fit using the Levenberg-Marquardt algorithm. Black lines show the 5^th^, 25^th^, 50^th^, 75^th^, and 95^th^ percentiles of percentage error in the estimated values for each parameter, with the light and dark shaded areas representing the 5-95 and 25-75 percentile ranges respectively. Vertical red lines and shaded areas are used to show the distribution of R_2_* values in the data, with lines representing percentiles (5^th^, 25^th^, 50^th^, 75^th^, and 95^th^) and shaded areas representing percentile ranges (5-95 and 25-75 for light and dark areas respectively).

Figure 3: A) Figure showing the mean GM correlation null distribution (obtained with phase-randomised MAP data) and mean GM correlation (with uncorrupted phase information). All subjects show a mean GM correlation that is more than 3 standard deviations outside the null distribution. B) Figure showing individual subject 3T BOLD – MAP correlations and corresponding p-values determined using permutation testing with phase-randomized MAP time series.

Figure 4: A) Group mean effect size of MAP on unfiltered BOLD signal (%BOLD / mm Hg) and threshold z-score.
